# Perspectives of older adults, caregivers, healthcare providers on frailty screening in primary care: a systematic review and qualitative meta-synthesis

**DOI:** 10.1186/s12877-022-03173-6

**Published:** 2022-06-03

**Authors:** Jiahui Nan, Yunzhu Duan, Shuang Wu, Lulu Liao, Xiaoyang Li, Yinan Zhao, Hongyu Zhang, Xianmei Zeng, Hui Feng

**Affiliations:** 1 Xiangya School of Nursing, Central South University, Changsha, Hunan China; 2 Xiangya-Oceanwide Health Management Research Institute, Central South University, Changsha, Hunan China

**Keywords:** Frailty screening, Primary care, Qualitative meta-synthesis

## Abstract

**Background:**

Screening is often recommended as a first step in frailty management. Many guidelines call to implicate frailty screening into practice in the primary care setting. However, few countries or organizations implement it. Understanding and clarifying the stakeholders’ views and issues faced by the implementation is essential to the successful implementation of frailty screening. However, the systematic review on stakeholders’ views of frailty screening in primary care is decidedly limited. Our objective was to explore the perspective of older adults, caregivers, and healthcare providers on frailty screening and determine the enablers and barriers to implementing frailty screening in primary care.

**Methods:**

A systematic search of six databases and other resources was conducted following JBI’s three-step search strategy. The search resulted in 7362 articles, of which 97 were identified for further assessment according to the inclusion criteria. After the full-text screening, quality assessment and data extraction were carried out using the tools from Joanna Briggs Institute (JBI). Moreover, reviewers used the approach of meta-aggregative of JBI to analyze data and synthesis the findings.

**Results:**

Six studies were included. A total of 63 findings were aggregated into 12 categories and then further grouped into three synthesized findings:1) capacity of healthcare providers and older adults; 2) opportunity in the implementation of frailty screening; 3) motivation in the implementation of frailty screening. These themes can help identify what influences the implementation of screening from the perspective of stakeholders.

**Conclusions:**

This meta-synthesis provides evidence on the barriers and enablers of frailty screening in primary care, from the aspects of psychological, physical, social, material, etc. However, stakeholder perspectives of frailty screening have not been adequately studied. More research and efforts are needed to explore the influencing factors and address the existing barriers.

**Supplementary Information:**

The online version contains supplementary material available at 10.1186/s12877-022-03173-6.

## Introduction

Increasing life expectancy leads to the rapid ageing of populations around the world [1]. By 2050, approximately 16% of the global population will be 65 years or older [2]. Frailty is a complex clinical syndrome characterized by marked vulnerability due to a decline in reserve and function across multiple physiologic systems [3, 4]. Systematic reviews indicated that the prevalence of frailty is 11% (ranging between 4.0 and 59.1) [5], and the global incidence of frailty was approximately 4.3% among community-dwelling older adults [6]. Older people with frailty may lead to adverse health outcomes, such as falls, fractures, hospitalizations, iatrogenic complications, early mortality, and lowered quality of life [7–12]. Given the increasingly high prevalence of frailty and its strong association with numerous adverse health outcomes, frailty is one of the most severe global public problems we will face [13]. Research reported that frailty could be prevented and possibly reversed [14]. Therefore, strategies to prevent and manage frailty are paramount. Screening is often recommended as a first step in frailty management [13].

Screening is a process for evaluating the possible presence of a particular problem [15]. It differs from “assessment”; therefore, this review focused on “screening”. Screening can detect frailty at individual and societal levels and provide information for assisting general decision-making [3] and the implementation of interventions to prevent and reverse frailty [16]. Some studies have indicated that frailty could be reversed through an early screening followed by the appropriate intervention [17–19]. Furthermore, the International Conference of Frailty and Sarcopenia Research (ICFSR) Guidelines strongly recommend that all adults aged 65 years and over be offered frailty screening [20].

Primary Care, as the first point of contact for patients, is the appropriate setting for addressing most of the population’s healthcare need [21]. Furthermore, primary care uses a comprehensive and patient-centered approach [22]. It can screen naturally older adults early in their trajectory and be more likely to be amenable to intervention [18, 23]. Therefore, primary care appears to be the most logical place to screen and manage frailty than other health-care system settings [22, 24]. Some national policies (England [25] and Australia [26]) and guidelines [3, 18, 20, 24, 27, 28] have recently called to implicate frailty screening in primary care settings. For example, the General Practice Contract for England (NHS England, 2017b) included for the first time a contractual requirement in 2017–2018, which requires general practitioners to detect patients aged ≥ 65 years with moderate to severe frailty [25]. However, to our knowledge, few countries or organizations implement it, except England. Many individuals who are frail or at risk of frailty may go undetected [29]. Due to the significant challenge for healthcare systems, many countries have low readiness to address it and considerable variation in access to resources [30]. Some obstacles hinder the implementation, including lack of public awareness of frailty, lack of treatment pathways, and the acceptability of older persons to screening [30, 31]. Studies pointed out that for frailty screening to move towards implementation, screening approaches must be acceptable to the older adults [32], and healthcare providers must see the screening benefit [33, 34]. Moreover, family caregivers play an essential role in engaging and empowering frail older adults, and they have the potential to either aid or hinder frailty screening [35, 36]. Therefore, understanding and clarifying the stakeholders’ views and issues faced by the implementation is essential to the successful implementation of frailty screening [37]. Qualitative research seeks to understand and interpret personal experience, behaviours, and social text to explain the phenomena of interest [38] and explore why an intervention is not adopted despite evidence of its effectiveness [39].

The previous reviews focused on the concept of frailty, frailty interventions, and the frailty screening instruments in primary care [16, 32, 40–43]. However, little is known about the frailty screening from stakeholders’ perspectives. One recent published protocol wants to explores the knowledge and attitude of healthcare professionals to frailty screening and identify the barriers and facilitators affecting the adoption of frailty screening in primary care from the perspective of healthcare professionals [44]. However, exploring the different perspectives of older adults, caregivers, and healthcare providers (HCPs) can more increase mutual understanding and communication [37]. The systematic review on stakeholders’ views of frailty screening in primary care is decidedly limited to the best of our knowledge. This meta-synthesis focuses on key stakeholders’ (older adults, caregivers, HCPs) views of frailty screening and synthesis of qualitative studies to analyze the factors facilitating and hindering the implementation of frailty screening in primary care. Moreover, providing an opportunity to facilitate the translation of research into clinical practice.

## Methods

### Research design

This qualitative systematic review was performed by the process and principles recommended by the Joanna Briggs Institute (JBI) approach [45]. The JBI uses a meta-aggregative approach to the synthesis of qualitative evidence. Meta-aggregation is a method that mirrors the accepted conventions for systematic review whilst holding to the traditions and requirements of qualitative research (it aggregates findings into a combined whole that is more than the sum of the individual findings in a way that is analogous with meta-analysis) [46]. It is sensitive to the specific characteristic of qualitative research [47] and enables generalized synthesized statements in the form of ‘lines of action’ to guide practitioners and policymakers [48]. This review was guided by the Enhancing Transparency in Reporting the Synthesis of Qualitative Research (ENTREQ) guidelines to report [49]. The systematic review protocol is registered with PROSPERO: CRD42021245807.

### Search strategy

This review followed JBI’s three-step search strategy. First, a search has been undertaken, followed by analyzing the text words contained in the title MEDLINE and CINAHL and abstract, and of the index terms used to describe the article. Secondly, a comprehensive search strategy was conducted to seek all available studies. We searched the following six databases for the earliest available date to June 2021: CINAHL(EBSCOhost), PubMed (OvidSP), Embase (Ovid), Scopus, Web of Science and PsycINFO. Third, the reference list of all studies selected for critical appraisal will be searched for additional studies. Lastly, reviewers hand searched the database to acquire more studies. The research was limited to the English Language. Moreover, the full search strategy is provided in Additional file 1.

### Eligibility criteria and study selection

The inclusion criteria were chosen according to the PICo review [45]: (a) qualitative primary studies or extracted qualitative data in mixed methods; (b) studies focus on the perspectives of frailty screening with older adults, caregivers, and HCPs. We included informal caregivers, including spouses, children, relatives, and other untrained caregivers. Regarding HCPs, healthcare professionals working in primary settings were included, such as general practitioners, nurses, etc. (c) the context was the primary care setting. We excluded research that only used quantitative methods.

Following the search, all citations were imported into Endnote version X9, and duplicates were removed. We initially screened the title and abstract according to inclusion criteria. Furthermore, we retrieved the selected studies’ full text for assessment to ensure they met inclusion criteria. Two reviewers (NJH and DYZ) undertook each study selection process independently, and there were no disagreements.

### Quality assessment

The Joanna Briggs Institute Qualitative Assessment and Review Instrument (JBI-QARI) was used to assess the methodological quality of the included papers by two reviewers independently [45]. We established a cut-off point of six of the ten questions answered as “yes” to ensure the included high-quality studies [50]. Any disagreements between the two reviewers (NJH and DYZ) were resolved by discussion; when necessary, find a third reviewer (LLL) on the team.

### Data extraction and synthesis

Qualitative data will be extracted from papers included in the review using the standardized data extraction tool from JBI-QARI. The first author extracted relevant data from the six studies. The data extracted will include specific details about the populations, context, culture, geographical location, study methods, and the phenomena of interest relevant to the review question and specific objectives. The two reviewers (NJH and DYZ) carefully read the included articles, appraised and attributed all qualitative findings a level of credibility by two reviewers according to the following criteria: (1) unequivocal (U)-findings accompanied by an illustration that is beyond reasonable doubt and therefore not open to challenge; (2) credible (C)-findings accompanied by an illustration lacking clear association with it and therefore open to challenge; (3) unsupported (Un)-findings are not supported by the data. There is no disagreement with the credibility level of each finding.

This review uses a meta-aggregative approach to the synthesis of qualitative evidence. It contains a three-stage process that was conducted by NJH and DYZ. First, the reviewers extracted all findings from all included articles and established a level of credibility. Secondly, developing categories for findings that are sufficiently similar, with at least two findings per category. Only unequivocal and credible findings will be included in the aggregation. Not-supported findings will be presented separately. Finally, developing one or more synthesized findings of at least two categories, looking for conceptual or descriptive commonality. After synthesizing, reviewers used the ConQual tool to evaluate the confidence of the synthesized findings [51].

## Results

### Search results

**Fig. 1:**
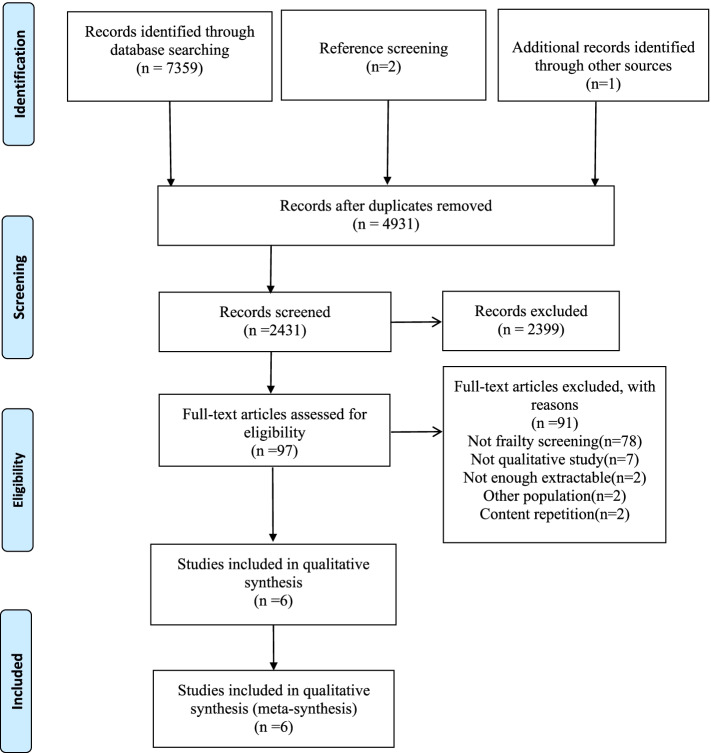
PRISMA 2009 Flow Diagram for identification and selection of included studies

The initial searches located 7362 references and removes 2431 duplicates by software. After screening the title and abstract, 97 papers were for further assessment. Finally, six papers were included in this review after reading the full text. Figure 1 presents a flow chart for the selection of the articles.

### Characteristic of studies

Among the six studies included in this review, four [29, 34, 52, 53] were qualitative research, and two [54, 55] were mixed-method studies. Five [29, 34, 52, 53, 55] were the journal articles, and one [54] is a master’s thesis. Of the six studies, two [52, 53] were from Australia, one [55] was from England, one [54] was from Ireland, one was from Canada, [29] and one [34] was conducted in three European Union countries (Ireland, Poland, and the United Kingdom). All included papers were published between 2017 and 2021. Detailed data are presented in Additional file 2.

### Methodological quality

All included studies scored 7–9. And no included papers were excluded. Among the six studies, all papers met criteria 2, 3, 5, 8, 9, and 10. However, five papers were unclear in the philosophical perspective (criteria 1), as papers did not clearly state the philosophical or theoretical premise on which the study is based. Table 1 provides the results of the quality assessment.

**Table 1:** Critical appraisal results for included studies using the JBI-Qualitative Critical Appraisal Checklist

**Citation**	**Shaw, R. L. et al.** [34]	**Boland, M. et al.** [54]	**C Ambagtsheer, R. et al.** [30]	**Mulla, E., et al.** [55]	**Archibald, M. M. et al.** [53]	**Van Damme, J. et al.** [29]
Q1^a^	U	U	U	U	Y	U
Q2^b^	Y	Y	Y	Y	Y	Y
Q3^c^	Y	Y	Y	Y	Y	Y
Q4^d^	Y	U	Y	Y	U	Y
Q5^e^	Y	Y	Y	Y	Y	Y
Q6^f^	N	N	N	Y	N	Y
Q7^g^	Y	Y	U	Y	U	U
Q8^h^	Y	Y	Y	Y	Y	Y
Q9^i^	Y	Y	Y	Y	Y	Y
Q10^j^	Y	Y	Y	Y	Y	Y
Score	8	7	7	9	7	8

^a^Is there congruity between the stated philosophical perspective and the research methodology?

^b^Is there congruity between the research methodology and the research question or objectives?

^c^Is there congruity between the research methodology and the methods used to collect data?

^d^Is there congruity between the research methodology and the representation and analysis of data?

^e^Is there congruity between the research methodology and the interpretation of results?

^f^Is there a statement locating the researcher culturally or theoretically?

^g^Is the influence of the researcher on the research, and vice- versa, addressed?

^h^Are participants, and their voices, adequately represented?

^i^Is the research ethical according to current criteria or, for recent studies, and is there evidence of ethical approval by an appropriate body?

^j^Do the conclusions drawn in the research report flow from the analysis, or interpretation, of the data?

### Meta-synthesis of qualitative data

Sixty-three findings were extracted from six papers. The findings were extracted from older adults and HCPs, and reviewers cannot extract valuable data from caregivers. Most of the findings were rated ‘unequivocal,’ two were rated ‘credible,’ and two were rated ‘unsupported.’ The unsupported finding did not include in the aggregation. Therefore, the 61 findings were aggregated into 12 categories according to the similarity of meanings, then developed three synthesized findings from the categories. Table 2 presents the themes of meta-synthesis. Moreover, the result of the meta-synthesis is shown in Additional file 3.

**Table 2:** Themes of Meta-synthesis

Synthesized finding	Category	Shaw, R. L. et al., [34]	Boland, M. et al., [54]	C Ambagtsheer, R. et al., [30]	Mulla, E., et al., [55]	Archibald, M. M. et al., [53]	Van Damme, J. et al., [29]
Capacity of healthcare providers and older adults	Lack of frailty and screening knowledge and skills among healthcare provider			√	√		√
	Lack of perception of frailty and screening among older adults	√				√	
Opportunity in the implementation of frailty screening	Lack of a proper tool				√	√	√
	Lack of an appropriate screening pathway	√	√	√		√	√
	Constructing a trustful relationship					√	√
	Conducting frailty screening by a sensitive approach					√	
	Involve the multidisciplinary team		√				√
Motivation in the implementation of frailty screening	Lack of support evidence of screening effectiveness				√		√
	Positive attitude toward frailty screening among healthcare provider		√	√	√		√
	Healthcare providers perceived benefits of screening		√	√			
	Older adults fear and escape frailty					√	√
	Older adults question the community’s insufficient resources	√				√	

### Synthesized finding1: Capacity of healthcare providers and older adults

It is essential to recognize that stakeholders’ capability influences the implementation of frailty screening. Education and training are needed to improve healthcare professionals’ knowledge and skills and improve older adults’ perception.

Healthcare providers lack knowledge and skills for frailty and screening. They have an incomplete understanding of frailty and regularly apply an intuitive screening through several typical warning signs and clinical judgment.

*I think … you know, you can assess people’s frailty within four seconds of looking at them, really ….*

*… within the first couple of seconds you know what’s going on; you can see how long it takes them to get up, you can see if they use the armrest, you see if they don’t need to do that, if they’ve got a walker or a frame or a stick, or if someone’s helping them, if they’re stooped over, their pace within the room. (C Ambagtsheer, R. et al.,* [30]*, P429)*

Some of those who used frailty screening tools are also doubtful about how to apply them, and it hampered HCPs’ ability to identify and manage older people.

*I have been a GP thirty-five years-plus and these are new terms to us for our understanding … who is severely frail and who is moderately frail.’ (GP4, female, partner, late career)(Mulla, E., et al. ,* [55]*, P607)*

Older adults lack of perception of frailty and screening. They think frailty is not preventable and query the necessity of formal frailty screening.

*When asked if frailty was preventable, there was a sense among participants that it may be possible to delay the symptoms of frailty and maintain wellbeing for some length of time but that it was not possible to prevent the inevitable, ‘to stave off the evil day?!’, i.e. that older adults will become frail if they continue to live. (Shaw, R. L. et al.,* [34]*, P1243)*

*This was exemplified by one community-dwelling participant who stated, ‘I think people would know without having to do a survey whether they were frail or not’ (FG3, female).(Archibald, M. M. et al. ,* [53]*, P229)*

### Synthesized finding2: Opportunity in the implementation of frailty screening

It must be noted that opportunity is an essential factor influencing the implementation of frailty screening. An awareness of the factors that reduce opportunities to implement frailty screening, including lack of a proper tool and lack of a clarity implementation pathway, is important. Moreover, a sensitive implementation approach and communication are conducive to creating a trusting relationship, and it can facilitate participation in frailty screening among older adults. Involving the multidisciplinary team can also promote the implementation of screening.

Many formal screening tools have been developed for HCPs, and each one has different properties and characteristics. Older people and HCPs think these tools lack sensitivity, specificity, logic, and accuracy.

*There is definitely under-identification of people who are frail but don’t necessarily have lots of long-term conditions.’*

*‘Having undertaken quite a lot of reviews of patients who are tagged by the electronic Frailty Index as being severely frail, we found out that, actually, they are either not frail at all or moderately frail.’ It would throw up surprising people as having [a] high frailty index [score] … we looked at the top one hundred patients and I would think [of] at least twenty that we saw, there is no way they should be on this index.’ (Mulla, E., et al. ,* [55]*, P608)*

Healthcare providers recommend that there be found and use a proper tool for more consistency. This proper screening tool should be multifactor, including functional ability, nutrition, psychological, pharmacy, and pain, and quick and easy to administer.

*HCP: “one of the things that frustrates me is when there is no cognitive screening…I’m big on cognitive screening…I don’t care if they’re here for a non-cognitive reason. I want to know what their cognition is like because maybe they are here because their falling and maybe that’s because a person is taking a blood pressure pill twice a day instead of once a day and maybe that’s because they have dementia.” (Van Damme, J. et al. ,* [29]*, p.28)*

Participants reported that the screening pathway was unclear. There is a lack of a consensus on when screening is best applied and the screening frequency. Moreover, it needs the pathway to analyze the meaning of results and provide action to address the needs.

*PT1: I don’t think there’s much point in implementing something like this (EFS), into an assessment, unless we have a pathway to follow through on it (Boland, M. et al.,* [54]*, P36)*

Healthcare providers recommend that it is crucial to understand the screening purpose and context. They think screening should be distinguished between universal screening and targeted screening according to the purpose.

*One is detecting at risk … versus one already with a condition. … That’s more an assessment of how bad it is; the other one is … a predictive value about where this may be going ... (C Ambagtsheer, R. et al.,* [30]*, P429-P430)*

Considering that the term frailty is perceived negatively by older adults, it is emphasized that providers need a sensitive approach to implementing screening. Some factors that administer a frailty-screening tool; and the length, terminology, and structure of the tool itself were regarded as necessary for sensitive screening.

*As one participant expressed, ‘if the person knows that they’re five out of ten, does that then say to them okay, well you know I don’t have to try. You know I’m on my way out sort of thing ... that’s more of a deterrent’.*

*Shorter tools were preferred to avoid giving up, or ‘feeling agitated and upset and nervous’ (e.g. with an hour-long test). (Archibald, M. M. et al.* [53]*, P230)*

Constructing a trusting relationship between HCPs and older adults is conducive to implementing a successful frailty screening. Providers identified their role in providing information. Healthcare providers thought some older people would potentially regard screening as valuable through proper communication with providers.

*As one community-dwelling male expressed, ‘if the tool could diagnose what’s going to happen to me, then I’d be better placed to go forward’ (Archibald, M. M. et al.,* [53]*, FG2).*

*OA: “you want to know sort of how it would affect your physical health and how it would progress that you would maybe ugh, you’d want to like do, manage things for yourself as long as you could.” (Van Damme, J. et al.,* [29], Additional File1)

Participants suggested that involving the multidisciplinary team can facilitate implementing frailty screening. Frailty is a multifactor clinical syndrome. Multidisciplinary teams (MDT), as an integrated management approach, provide an excellent opportunity to screen. Older adults also support the use of MDT for their health.

*PT6:” Frailty is multifactorial I suppose, so you’d need a multidisciplinary approach. So we can definitely help as physios but we need to involve GPs, nurses, family.”(Boland, M. et al.,* [54]*, P35)*

*OA: “this is where, you know, these clinics that some doctors have set up, are an excellent idea. Because you’ve got a dietician, you’ve got a physiotherapists, you’ve got an occupational therapist, you know you’ve got all these people, and so you know the doctor can call on all these people for extra assistance.” (Van Damme, J. et al.,* [29], Additional File1)

### Synthesized findings 3: Motivation in the implementation of frailty screening

Motivation is those brain processes that energize and direct behaviour in implementing frailty screening. Healthcare providers’ positive attitude and the belief in the benefits of screening facilitate the implementation. Factors that hinder the implementation include the lack of supportive evidence of screening effectiveness, older adults’ fear of frailty, and doubt about community insufficient resources.

Proactivity is reflected in the fact that most HCPs hold a positive view of frailty screening and realize its benefits. Providers think they can accept it and recognize the benefits of formal screening, which can identify and address frailty early and help elders in a holistic approach.

*‘In principle, it is a really good idea … What I think it does and the reason I think it does have value is that it helps us identify cohorts of patients who are potentially at risk and who will benefit.’*

*Prevention is better than cure, so if you identify somebody that would be a good place to start.’ (Mulla, E., et al.,* [55]*, P.607)*

Lack of support evidence of screening effectiveness may hinder some providers from implementing screening. The proof that screening led to improved older adults’ frailty is lacking, and the HCPs think little could be done to influence frailty.

*‘We can identify and label people with diseases, but actually if there is not much you can do about it … I am not sure who is happier, or if anybody is.’ (Mulla, E., et al.,* [55]*, P607)*

Older adults fear frailty and even escape it, limiting them from participating in screening. They regard frailty and screening with fear and apprehension, and they do not want to know whether they are frail.

*‘am I frail? Will you test me? There’s no way you’re going to go in and say that and if you answer all their questions right which you know as well as anybody, they can answer questions really good. Go out the door and say something stupid but they can...the GP is not going to pick it up’ (Archibald, M. M. et al.,* [53]*, P229)*

Older adults think the formal screening should be consultative and inform specific actions, but they question whether the community has sufficient resource to provide services.

*It would be very expensive. Access to health care would be improved. (Shaw, R. L. et al.,* [34]*, P1244)*

*‘we need someone to take up on the people that are frail and is there the resources available to fix it. I doubt that’.(Archibald, M. M. et al.,* [53]*, P230)*

### ConQual ‘Summary of Findings’

This review using the system ‘ConQual’ [51] to rate the confidence of synthesized qualitative findings. The ConQual summary of findings is shown in Table 3.

**Table 3:** ConQual summary of findings

Systematic review title: Perspectives of older adults, caregivers, healthcare providers on frailty screening in primary care: a systematic review and qualitative meta-synthesis Population: older adults, caregivers, and healthcare providers Phenomena of interest: the perception of frailty screening Context: in primary care				
Synthesized finding	Type of research	Dependability	Credibility	ConQual score
Capacity of healthcare providers and older adults It is important to recognize that stakeholders’ capability exerts influence on the implementation of frailty screening. Need education, training, enablement to improve healthcare professionals’ knowledge and skills, and further the perception of frailty in the elderly	Qualitative	Downgrade 1 level^a^	remains unchanged	Moderate
Opportunity in the implementation of frailty screening It must be noted that opportunity is an essential factor influencing the implementation of frailty screening. An awareness of the factors that reduce opportunities to implement frailty screening, including lack of a proper tool and lack of a clarity implementation pathway, is important. Moreover, a sensitive implementation approach and communication are conducive to creating a trusting relationship, and it can facilitate participation in frailty screening among older adults. Involving the multidisciplinary team can also promote the implementation of screening	Qualitative	Downgrade 1 level^a^	Downgrade 1 level^b^	Low
Motivation in the implementation of frailty screening Healthcare providers’ positive attitude and the belief in the benefits of screening facilitate the implementation. Factors that hinder the implementation include the lack of supportive evidence of screening effectiveness, older adults’ fear of frailty, and doubt about community insufficient resources	Qualitative	Downgrade 1 level^a^	remains unchanged	Moderate

^a^Downgraded one level due to common dependability issues across the included primary studies (the majority of studies did not present a statement locating the researcher culturally or theoretically, and there was no acknowledgment of their influence on the research)

^b^Downgraded one level to a mix of unequivocal and credible findings

## Discussion

This systematic review identified six qualitative studies from diverse countries on primary care and included the views of different stakeholders: older adults and health care providers. Two papers involved caregivers but did not extract enough data to be valuable. This review developed three themes by systematically synthesizing qualitative research: stakeholder capacity, opportunity in the implementation of frailty screening, and motivation in the implementation of frailty screening. Furthermore, these themes reveal crucial factors that promote or hinder the implementation of frailty screening in primary care. It is timely, with the strongly recommended frailty screening in the primary care setting and the lack of implementation [20].

Stakeholders’ capacity is the crucial factor that affects the successful implementation of frailty screening in primary care. This finding is consistent with the other clinical settings; for example, the lack of knowledge and understanding about frailty and screening was a significant barrier in acute care settings and hospitals [56, 57]. Frailty can be positioned as a long-term condition because it shares similar characteristics as other long-term conditions, such as diabetes and arthritis [58]. Therefore, effective frailty prevention and management need to educate older people, their caregivers, and HCPs [42, 59], and some researchers have aware of these needs [58].In 2018, the Health Education England and NHS England developed a framework of frailty core capacity of knowledge and skills for educating frail older people, their caregivers, and HCPs that contained four domains, fourteen capabilities [60], including a) understanding, identifying, and assessing frailty, b) person-centered collaborative working, c) managing frailty, d) underpinning principles. Moreover, some HCPs expressed their preference for online training formats (either on an individual basis or in small groups), the opportunity for a placement in geriatric care units, and practical skills demonstration [42]. In addition, patient education is becoming an essential component of frailty management [58]. Researchers suggest that education programs should involve family caregivers, specific objects, and materials suitable for older adults [58]. Raising awareness about frailty among HCPs and the general population is the first step in health systems’ response to frailty and maintaining self-capacity for increasing older people [61, 62].

It has been found that challenges mainly focus on the field of opportunity. Healthcare professionals indicated a lack of a proper tool in screening and expressed their concerns about the tool’s accuracy, sensitivity, and specificity. Dozens or hundreds of frailty tools have been developed with the increasing interest in frailty [18]. Many experts studied to find a suitable tool used in primary care [63–65]. However, no selected frailty tools built so far could be used as a screening tool because of the inadequate sensitivity or specificity [66]. Studies recommend three features that the appropriate frailty screening tools used in the primary care setting should meet: multidimensional structure, quick and easy use, and high accurate risk prediction of adverse outcomes [67]. More than the proper tool, defining a common pathway followed by the different actors involved matters [67]. The lack of a screening pathway is a barrier to implementing frailty screening, as HCPs in confused about when and how often the screening should be initiated, the meanings of the results, and corresponding actions. ICFSR guidelines recommend offering frailty screening for all adults aged 65 years [20]. An article mentioned using the Integrated Care for Older People (ICOPE) to manage frailty and suggested the screening frequency was four months [68]. As for the screening, the pathway could be a two-step process to manage frailty-implementing multidimensional screening for all individuals and assessing frail ones [69]. In 2013, the Netherlands carried out mass screening using the PERSSILAA screening pathway, a two-step annual screening program, to identify older adults at risk of frailty [70]. Older adults first completed a self-screening questionnaire to screen their general health status, and then those who were at risk of becoming frail were invited for a face-to-face assessment. While the England General Medical Services (GMS) contract screens people over 65 to identify patients who may be living with severe or moderate frailty by using a two-step contract process to manage: step 1 identify potential frailty using eFI, step 2 apply clinical judgment to confirm or further consideration [25]. However, there is no recommended clarity pathway to describe screening details and what needs to be action corresponding to the screening score. That requires more research. A trustful communication relationship and a sensitivity screening approach can help alleviate the potential burden of labelling older adults as frail and negatively influencing patients’ behaviours [71]. In addition, the involvement of an MDT can enhance the effectiveness of frailty screening [56]. Frailty is a multidimensional syndrome, so management in primary care requires an integrated approach [24]. Primary care providers need to collaborate with MDT, which involves geriatricians, allied health professionals, caregivers, and the patient themselves to ensure the delivery of patient-centered integrated care [68]. While the frailty tool and the screening pathway are not new, this review shows the stakeholders’ need for a proper tool and the clarity pathway.

Recognizing motivation in screening is very important in promoting successful screening implementation. Healthcare providers hold a positive attitude towards the implementation of screening. They believe formal screening can identify frailty or the risk of frailty early and increase their holistic awareness of the patient [72]. Even though more research demonstrated the role of frailty as a predictor of adverse outcomes such as mortality, functional decline, and length hospital stays [10–12]. There is a lack of current evidence to support that the screening could improve frailty management [20], hindering the providers from carrying out frailty screening. Insufficient resource in the community is another obstacle [73]. Older adults question the community’s adequate resources to implement screening and provide related services. Indeed, most countries underinvest in primary care [74]. For instance, primary care accounts for 5%-7% of total health care spending despite primary health care being the largest speciality in the U.S. health system [75]. WHO calls for more investment in primary care and indicates “funding and allocation of resources” as one of the core strategic levels [76]. Therefore, we recommend that countries invest more in primary health care to facilitate the implementation of frailty screening. Moreover, findings from this review highlight that a barrier is that older adults fear frailty and screening and even escape the problem. It is reported that older adults associated the term “frail” and “frailty” with negative age-related stereotypes [41] and vehemently avoided discussing frailty [77]. It has been on the agenda to reconsider the need to adapt to frailty for several years [78]. The World Health Organization (WHO) proposed “intrinsic capacity” to describe the individuals’ physical and cognitive health and overall functional ability in the World Report on Ageing and Health as a positive term for healthy ageing in 2015 [1].

This review provides researchers insights into the perspectives of stakeholders on frailty screening and enhances researchers’ understanding of the potential factors that may influence frailty screening practice. However, this review has some limitations. Firstly, we only included research reported in English. The language limitation may lead to overlooking some studies. Secondly, we only included six articles, so the information may not be sufficient. Furthermore, because of the limitations of included articles, we did not conduct a stratified analysis according to the types of healthcare providers. Thirdly, our review could not extract valuable caregivers’ data from the included studies. Finally, all the studies were conducted in developed countries, so the findings may not be applicable elsewhere.

## Conclusion

The meta-synthesis has provided synthesized qualitative evidence that can guide the implementation of frailty screening in primary care. Three themes identified in the systematic review are stakeholder capability, opportunity in the implementation of frailty screening, and motivation in the implementation of frailty screening. These themes can help identify what influences the implementation of screening. In addition, more high-quality quantitative and qualitative papers are needed to explore stakeholders’ perspectives in different countries (especially in middle-income and low-income countries) to establish evidence or strategy to implement frailty screening in primary care successfully.

## Implications for research

Recommendations for practice arising from the review are provided in Additional file 4. Per guidelines has been assigned a grade according to the JBI Grade of Recommendation [79]. Grade A is a strong recommendation, while Grade B is a weak recommendation. Research recommendations are provided below. Tt is necessary to explore the effectiveness of frailty screening, identify a proper instrument and a screening pathway to strengthen the evidence on implementing frailty screening in primary care. And there is a need for researches to focus on designing, developing, and implementing different education programs about frailty.

## Supplementary Information

**Additional file 1.** Search Strategies.

**Additional file 2.** QARI data extraction of included studies.

**Additional file 3.** Results of meta-synthesis.

**Additional file 4.** Recommendation for practice.

## Data Availability

All data generated or analysed during this study are included in this published article [and its supplementary information files].

## Abbreviations

JBI: Joanna Briggs Institute
JBI-QARI: Joanna Briggs Institute Qualitative Assessment and Review Instrument
ICFSR: The International Conference of Frailty and Sarcopenia Research Guidelines
HCPs: Healthcare providers
ENTREQ: Enhancing Transparency in Reporting the Synthesis of Qualitative Research guidelines
MDT: Multidisciplinary teams
ICOPE: The Integrated Care for Older People
GMS: The England General Medical Services
WHO: World Health Organization
