# Supplementary material for: Perspectives of older adults, caregivers, healthcare providers on frailty screening in primary care: a systematic review and qualitative meta-synthesis
Source: BMC Geriatr. 2022 Jun 3;22:482. doi: 10.1186/s12877-022-03173-6 (PMC9166584; doi:10.1186/s12877-022-03173-6)
Supplement: Supplementary file 1 — Additional file 1. Search Strategies. [file 12877_2022_3173_MOESM1_ESM.docx]

**Additional file 1: Search Strategies**

Search strategies used for finding qualitative research articles about the perception of key stakeholders (older adults, caregivers, and healthcare providers) on frailty screening in primary care. Number of retrieved articles is given in the right-hand column.

**Pubmed**

| #1 | frailty[Mesh] | 4535 |
| --- | --- | --- |
| #2 | frail*[Title/Abstract] | 26911 |
| #3 | frail*[Title/Abstract] AND (screen*[Title/Abstract] OR assess*[Title/Abstract] OR identif*[Title/Abstract] OR detec*[Title/Abstract]) | 14718 |
| #4 | #1 or #2 or #3 | 27209 |
| #5 | aged[Mesh] | 3258994 |
| #6 | Aged[Title/Abstract] OR elderly[Title/Abstract] OR older adults[Title/Abstract] OR older people[Title/Abstract] OR older patients[Title/Abstract] | 929694 |
| #7 | #5 or #6 | 3764884 |
| #8 | caregivers[Mesh] | 40041 |
| #9 | "Spouses"[Mesh] | 10633 |
| #10 | caregiver*[Title/Abstract] OR spouses[Title/Abstract] OR "family caregiver*"[Title/Abstract] | 81886 |
| #11 | #8 OR #9 OR #10 | 102607 |
| #12 | “health personnel” [Mesh] | 542580 |
| #13 | General practitioners[Mesh] | 8558 |
| #14 | Nurse practitioners[Mesh] | 18198 |
| #15 | "health personnel"[Title/Abstract] OR "healthcare provider"[Title/Abstract] OR "health care provider"[Title/Abstract] OR "healthcare professional"[Title/Abstract] OR "health care professional"[Title/Abstract] OR "health care worker"[Title/Abstract] OR "general practitioner*"[Title/Abstract] OR "nurse practitioner*"[Title/Abstract] OR "community nurs*"[Title/Abstract] | 94306 |
| #16 | #12 or #13 or #14 or #15 | 607970 |
| #17 | stakeholder*[Title/Abstract] OR "health consumer*"[Title/Abstract] OR health user*[Title/Abstract] | 48055 |
| #18 | #7 or #11 or #16 or #17 | 4369190 |
| #19 | Qualitative Research[Mesh] | 65328 |
| #20 | "Grounded Theory"[Mesh] | 2005 |
| #21 | "Health Services Research"[Mesh] | 177425 |
| #22 | "Observation"[Mesh] | 5856 |
| #23 | "Focus Groups"[Mesh] | 32453 |
| #24 | "Interview" [Publication Type] | 29776 |
| #25 | Qualitative research[Title/Abstract] OR Qualitative study[Title/Abstract] OR ethnography[Title/Abstract] OR phenomenology[Title/Abstract] OR "grounded theory"[Title/Abstract] OR hermeneutic*[Title/Abstract] OR "experience*"[Title/Abstract] OR narrative*[Title/Abstract] OR "action research"[Title/Abstract] OR observation*[Title/Abstract] OR "focus group"[Title/Abstract] OR interview*[Title/Abstract] OR "mixed method"[Title/Abstract] OR multimethod[Title/Abstract] | 2370366 |
| #26 | #19 or #20 or #21 or #22 or #23 or #24 or #25 | 2529002 |
| #27 | "Primary Health Care"[MeSH] | 171268 |
| #28 | "Residence Characteristics"[MeSH] | 70199 |
| #29 | "primary care"[Title/Abstract] OR "community"[Title/Abstract] | 646753 |
| #30 | #27 OR #28 OR #29 | 801161 |
| #31 | #4 and #18 and #26 and #30  Filters: English | 1621 |

**Embase(OVID)**

| #1 | 'frailty'/exp | 15719 |
| --- | --- | --- |
| #2 | frail*:ab,ti | 39148 |
| #3 | frail*:ab,ti AND (screen*:ab,ti OR detec*:ab,ti OR assess*:ab,ti OR identif*:ab,ti) | 22996 |
| #4 | #1 or #2 or #3 | 41196 |
| #5 | 'older adults'/exp OR 'older people'/exp OR 'aged'/exp | 3305452 |
| #6 | elderly:ab,ti OR 'older patients':ab,ti | 416902 |
| #7 | #5 OR #6 | 3415175 |
| #8 | 'caregiver'/exp | 90046 |
| #9 | 'spouse'/exp | 20975 |
| #10 | 'family caregiver':ab,ti | 2197 |
| #11 | #8 OR #9 OR #10 | 108610 |
| #12 | 'health care personnel'/exp OR 'general practitioner'/exp OR 'nurse practitioner'/exp OR 'community nurse'/exp | 1706865 |
| #13 | 'healthcare professional':ab,ti OR 'health care professional':ab,ti OR 'healthcare provider':ab,ti OR 'health care provider':ab,ti OR 'community care staff':ab,ti | 23840 |
| #14 | #12 OR #13 | 1715431 |
| #15 | 'stakeholder'/exp | 45 |
| #16 | 'health consumer*':ab,ti OR 'health user*':ab,ti | 1368 |
| #17 | #15 or #16 | 1413 |
| #18 | #7 or 11 or #14 or #17 | 5057032 |
| #19 | 'qualitative research'/exp OR 'grounded theory'/exp OR 'observational study'/exp OR 'action research'/exp OR 'ethnography'/exp OR 'phenomenology'/exp OR 'focus group'/exp OR 'interview'/exp OR 'mixed method'/exp OR 'multimethod study'/exp | 614361 |
| #20 | 'primary health care'/exp OR 'community'/exp | 260647 |
| #21 | 'primary care':ab,ti OR community:ab,ti | 809390 |
| #22 | #20 OR #21 | 886743 |
| #23 | #4 AND #18 AND #19 AND #22 AND [english]/lim | 677 |

**Web of science**

| #1 | TS=(frail*) | 46564 |
| --- | --- | --- |
| #2 | TS=("older adults" OR "older people" OR "older patients" OR "aged" OR "elderly") | 6409379 |
| #3 | TS=("caregiver*" OR "family caregiver*" OR "spouse*") | 159888 |
| #4 | TS=("health personnel" OR "healthcare provider" OR "health care provider" OR "healthcare professional" OR "health care worker" OR"community care staff" OR "general practitioner" OR "nurse practitioner*" OR "community nurs*") | 259863 |
| #5 | TS=("stakeholder*" OR "healthcare consumer*" OR "healthcare user*") | 158663 |
| #6 | #2 or #3 or #4 or #5 | 6839409 |
| #7 | AB=(qualitative or ethnography or phenomenology or "grounded theory" or hermeneutic* or "experience*" or "narrative*" or "action research" or observation or "focus group" or "interview*" or "mixed method" or multimethod) | 5431360——  4339973 |
| #8 | TS=("primary care" OR "community") | 1731900 |
| #8 | #1 and #6 and #7 and #8 | 1769 |
| #9 | Filter: language: English | 1661 |

**Scoups**

| 1 | ( TITLE-ABS-KEY ( frail* ) OR TITLE-ABS-KEY ( frail* AND ( screen* OR assess* OR detec* OR identif* ) ) ) | 40863 |
| --- | --- | --- |
| 2 | TITLE-ABS-KEY ( aged OR elderly OR "older patient*" OR "older adult*" OR "older people" ) | 5714146 |
| 3 | TITLE-ABS-KEY ( caregiver* OR family AND caregiver* OR spouse* ) | 139986 |
| 4 | TITLE-ABS-KEY ( "health personnel*" OR "health professional" OR "health care professional" OR "healthcare provider" OR "health care provider" OR "community care staff" OR "general practitioner*" OR "nurse practitioner" OR "community nurs*" ) | 475479 |
| 5 | TITLE-ABS-KEY ( stakeholder OR "health consumer*" OR "health user*" ) | 205167 |
| 6 | #2 or #3 or #4 or #5 | 6338280 |
| 7 | TITLE-ABS-KEY ( qualitative OR ethnography OR phenomenology OR "grounded theory" OR hermeneutic$ OR "experience$" OR "narrative$" OR "action research" OR observation OR "focus group" OR "interview$" OR "mixed method" OR multimethod ) | 5485636 |
| 8 | TITLE-ABS-KEY ( "primary health care" OR "primary care" OR "community" ) | 1896301 |
| 9 | #1 and #6 and #7 and #8 | 1469 |
| 10 | Limits: English | 1388 |

**CINAHL (EbscoHOST)**

| S1 | (MH "Frailty Syndrome") | 2836 |
| --- | --- | --- |
| S2 | (MH "Frail Elderly") | 7997 |
| S3 | TI ( frail* or (frail* and (screen* or assess* or detec* or identif*)) ) OR AB ( frail* or (frail* and (screen* or assess* or detec* or identif*)) ) | 14301 |
| S4 | S1 OR S2 OR S3 | 17708 |
| S5 | (MH "Aged+") | 867760 |
| S6 | TI ( aged or elderly or "older adult*" or "older people" or "older patients" ) OR AB ( aged or elderly or "older adult*" or "older people" or "older patients" ) | 347626 |
| S7 | S5 OR S6 | 1037893 |
| S8 | (MH "Caregivers") | 37315 |
| S9 | (MH "Spouses") | 11364 |
| S10 | TI ( caregiver* or "family caregiver*" or "spouse*" ) AND AB ( caregiver* or "family caregiver*" or "spouse*" ) | 14382 |
| S11 | S8 OR S9 OR S10 | 50823 |
| S12 | (MH "Health Personnel+") | 594455 |
| S13 | (MH "Physicians, Family") | 20820 |
| S14 | (MH "Nurse Practitioners+") | 23280 |
| S15 | TI ("healthcare professional" or "health care professional" or "healthcare provider" or "health care provider" or "health care worker" or "general practitioner*" or "nurse practitioner*" or "community care staff" or "community nurs*") OR AB ("healthcare professional" or "health care professional" or "healthcare provider" or "health care provider" or "health care worker" or "general practitioner*" or "nurse practitioner*" or "community care staff" or "community nurs*") | 50806 |
| S16 | S12 OR S13 OR S14 OR S15 | 624059 |
| S17 | TI ( "stakholder*" or "health consumer*" or "health user*" ) OR AB ( "stakholder*" or "health consumer*" or "health user*" ) | 1013 |
| S18 | S7 OR S11 OR S16 OR 17 | 1645151 |
| S19 | (MH "Primary Health Care") | 67108 |
| S20 | (MH "Communities+") | 52461 |
| S21 | TI ( "primary care" or "community" ) OR AB ( "primary care" or "community" ) | 300578 |
| S21 | S19 OR S20 OR S21 | 367080 |
| S22 | S4 and S18 and S21  Limiters‐Clinical Queries: Qualitative - High Sensitivity; Language-English | 1946 |

**PsycInfo（EBSCOhost）**

| S1 | DE "Health Impairments" | 2991 |
| --- | --- | --- |
| S2 | TI ( (frail* or (frail* and (screen* or assess* or detec* or identif*)) ) ) OR AB ( frail* or (frail* and (screen* or assess* or detec* or identif*)) ) ) | 4944 |
| S3 | S1 OR S2 | 6319 |
| S4 | TI ( aged or elderly or "older adult*" or "older people" or "older patients" ) OR AB ( aged or elderly or "older adult*" or "older people" or "older patients" ) | 358475 |
| S5 | DE "Caregivers" | 36312 |
| S6 | DE "Spouses" | 14792 |
| S7 | TI ( caregiver* or "family caregiver*" or "spouse*" ) OR AB ( caregiver* or "family caregiver*" or "spouse*" ) | 68246 |
| S8 | S5 OR S6 OR S7 | 86331 |
| S9 | (DE "Health Personnel") OR (DE "General Practitioners") | 28580 |
| S10 | TI ( "healthcare professional" or "health care professional" or "healthcare provider" or "health care provider" or "health care worker" or "general practitioner*" or "nurse practitioner*" or "community care staff" or "community nurs*" ) OR AB ( "healthcare professional" or "health care professional" or "healthcare provider" or "health care provider" or "health care worker" or "general practitioner*" or "nurse practitioner*" or "community care staff" or "community nurs*" ) | 17161 |
| S11 | S9 OR S10 | 40703 |
| S12 | DE "Stakeholder" | 4907 |
| S13 | TI ( "stakholder*" or "health consumer*" or "health user*" ) OR AB ( "stakholder*" or "health consumer*" or "health user*" ) | 1002 |
| S14 | S12 0R S13 | 5904 |
| S15 | S4 OR S8 OR S11 OR S14 | 469953 |
| S16 | DE "Primary Health Care" | 23357 |
| S17 | DE "Community Health" | 3614 |
| S18 | TI ( "primary care" or "community care" ) OR AB ( "primary care" or "community care" ) | 34046 |
| S19 | S16 OR S17 OR S18 | 43618 |
| S20 | AB (qualitative OR ethnograph $ OR phenomenology $ OR “grounded theory” OR hermeneutic$ OR “experience$” OR narrative$ OR “action research” OR observation$ OR “focus group$” OR interview$ OR “mixed method” OR “multimethod”) | 845414 |
| S21 | S3 and S15 and S19 and S20  Limits-Language: English | 66 |

**TOTAL FOUND:7359**
