# Supplementary material for: Perspectives of older adults, caregivers, healthcare providers on frailty screening in primary care: a systematic review and qualitative meta-synthesis
Source: BMC Geriatr. 2022 Jun 3;22:482. doi: 10.1186/s12877-022-03173-6 (PMC9166584; doi:10.1186/s12877-022-03173-6)
Supplement: Supplementary file 2 — Additional file 2. QARI data extraction of included studies. [file 12877_2022_3173_MOESM2_ESM.docx]

**Additional file 2: QARI data extraction of included studies**

| **Author(year)** | **Methodology** | **Method** | **Phenomena of interest** | **Setting** | **Geographical** | **Cultural** | **Participants** | **Data analysis** | **Author^’^s conclusion** | **Reviewer^’^s comments** |
| --- | --- | --- | --- | --- | --- | --- | --- | --- | --- | --- |
| 1.Shaw, R. L. et al. (2017) | Qualitative study | Focus group and individual interview | European stakeholders’ accounts of the acceptability and feasibility of frailty screening and prevention | Three European Union countries | Italy, Poland and the United Kingdom | European Union | Frail and non-frail older adults; family care-givers and health care professionals. | Thematic analysis (Braun and Clark2006) | Frailty is a complex phenomenon with multifaceted component parts. Frailty screening could be implemented as a triage process, facilitating referral into appropriate care and treatment services for people with specific, identified health needs, ensuring that therapeutic measures are applied in a timely manner while preventing pre-frail individuals from deteriorating into a frail condition | This article explored stakeholders’ experience of frailty and the meanings of frailty to individuals; determine their understanding, beliefs and views on the malleability of frailty through screening and prevention programmes. |
| 2.Boland, M. et al. (2018) | Mixed method | focus group interviews | explore the acceptability of the Edmonton Frail Scale to physiotherapists and the influence of frailty screening on clinical practice of physiotherapists working with older adults in Primary Care. | Community Healthcare Organisation Dublin North City and County, | North Dublin, Ireland | Ireland | eight physiotherapists | Thematic analysis(Braun and Clark2006) | The EFS was an acceptable and useful measure for physiotherapists. The results of this study demonstrate the importance of a holistic, multidisciplinary approach to frailty in Primary Care and the need for integration between HSCPs to improve the effectiveness of frailty screening | This article explored three domains.  1.To explore physiotherapists’ perspective of the role of frailty screening in primary care.  2.To explore physiotherapists; experience of utilizing the EFS to screen for frailty  3.To explore physiotherapists’ perspectives on the influence of frailty screening on clinical practice in the primary care setting. |
| 3.C Ambagtsheer, R. et al. (2019) | a qualitative descriptive design | Focus group；semi-structured interview | GPs’ perceptions, attitudes and experiences of frailty and frailty screening. | Adelaide, South Australia | Adelaide, South Australia | Australia | twenty and two General practitioners | Thematic analysis | Australian GPs may be open to a proactive approach to frailty assessment and treatment, given appropriate training and resources | Understand GPs’ perception and experience on frailty and frailty screening |
| 4.Mulla, E., et al. (2021). | A sequential mixed-methods | a semi-structured telephone interview. | GPs’ views on identifying frailty and offering interventions for those living with moderate or severe frailty. | in the East Midlands region of England | in the East Midlands region of England | England | eighteen general practitioners | Framework analysis | Proactively identifying and responding to frailty in primary care requires GP education, highly sensitive and specific risk-stratification tools, better access to interventions to lessen the impact of frailty, and adequate resourcing to achieve potential clinical impact. | Understand GPs’ perception on identifying frailty and offering interventions; identify drivers of GP behavior. |
| 5.Archibald, M. M. et al. (2021) | interpretive descriptive qualitative design. | focus groups | exploring older adults’ views on frailty screening | Community, and residential aged care settings | metropolitan South Australia | Australia | Thirty-nine older adults (22 community-based, 17 residential aged care) | inductive thematic analysis | the lack of public awareness of frailty and the nascent stage of frailty screening implementation | To explore older adults’ views on frailty screening |
| 6.Van Damme, J. et al. (2020) | a qualitative methodological approach | a qualitative methodological approach, | examined older adults’, caregivers’ and healthcare providers’ perspectives on frailty and frailty screening | southwestern Ontario, Canada | southwestern Ontario, Canada | Canada | older adults and caregivers (n= 14), healthcare providers (n = 14) | Thematic analysis | Older adults, caregivers and healthcare providers have similar perspectives regarding frailty; both identified frailty as multi-dimensional and dynamic. Healthcare providers need clear “next steps” to provide meaning to frailty screening practices, which may improve use of frailty-screening tools. | This article explored key stakeholders’ perspectives on frailty and frailty screening. Describe from the concept of frailty; frailty screening current practice; limitations in frailty screening; recommendations for frailty screening et al. |
