# Supplementary material for: Perspectives of older adults, caregivers, healthcare providers on frailty screening in primary care: a systematic review and qualitative meta-synthesis
Source: BMC Geriatr. 2022 Jun 3;22:482. doi: 10.1186/s12877-022-03173-6 (PMC9166584; doi:10.1186/s12877-022-03173-6)
Supplement: Supplementary file 3 — Additional file 3. Results of meta-synthesis. [file 12877_2022_3173_MOESM3_ESM.docx]

**Additional file 3: Results of meta-synthesis**

| **Finding** | **Category** | **Synthesized finding** |
| --- | --- | --- |
| Incomplete understanding of frailty. (U) | 1.Lack of frailty and screening knowledge and skills among healthcare provider | Synthesized finding1: Capacity of healthcare providers and older adults  It is important to recognize that stakeholders’ capability exerts influence on the implementation of frailty screening. Need education and training to improve healthcare professionals’ knowledge and skills, and further the perception of frailty in the elderly. |
| They regularly applied a type of rapid, intuitive screening when interacting with older patients. (U) |  |  |
| GPs already knew who is frail relying on a number of typical warning signs and could potentially readily choose them from a list without the need for formal tools(U) |  |  |
| Uncertainty about application of electronic tools. (U) |  |  |
| Often current screening practices involves the use of clinical judgement. (U) |  |  |
| Home visits were identified by healthcare providers as an opportunity to gather information to assist in assessing a patient. (U) |  |  |
| A large part of how providers currently assess patients is through a patient history. (U) |  |  |
| Providers use their own methods to determine the functional ability of clients, often based on more formal screens, but modified to suit their unique situational needs. |  |  |
| They think frailty is not preventable but can delay the symptoms. (U) | 2.Lack of perception of frailty and screening among older adults |  |
| older adults question the necessity of frailty screening. (U) |  |  |
| Stratification tools-Lack of sensitivity.(U) | 3.Lack of a proper tool | Synthesized finding2: Opportunity in the implementation of frailty screening  It must be noted that opportunity is an essential factor influencing the implementation of frailty screening. An awareness of the factors that reduce opportunities to implement frailty screening, including lack of a proper tool and lack of a clarity implementation pathway, is important. Moreover, a sensitive implementation approach and communication are conducive to creating a trusting relationship, and it can facilitate participation in frailty screening among older adults. Involving the multidisciplinary team can also promote the implementation of screening. |
| Stratification tools -Lack of specificity(U) |  |  |
| older adults question the logic of frailty screening(U) |  |  |
| Providers identified concerns with the accuracy of current screening tools as well. (U) |  |  |
| Factors that should be included in screening (U)  1.a baseline for screening  2.assessing functional abilities such as mobility, strength, and level of independence and pain  3.pharmacy  4.nutrition  5.psychological |  |  |
| Current screening tools (U) |  |  |
| The need for more consistency (U) |  |  |
| Inconsistent screening (U) |  |  |
| Tools need to be quick and easy to administer (U) |  |  |
| How screening could help older adults to maintain a positive outlook by showing them how to keep going with activities they enjoyed in a safe way that would not threaten their health. (U) | 4. Lack of an appropriate screening pathway |  |
| To be useful, screening had to be paired with access to services for older adults(U) |  |  |
| frailty screening needs to result in an action(U) |  |  |
| Provider’s main concern with frailty screening was a lack of understanding about what the results mean. Providers agree that identifying frailty was important, but screening is ineffective at articulating the implications of a score. Results should provide more meaningful information for patient（U） |  |  |
| Frailty as a worsening cycle of decline，GPs differed with respect to how quickly they perceived the general rate of decline to be, this perception was likely related to how frequently they saw the patients.(U) |  |  |
| lack of consensus for when universal screening should start(C) |  |  |
| The time of screening (U) |  |  |
| Screening pathway is not clear. (U) |  |  |
| Participants reported that only identifying frailty is not sufficient and that a pathway to address the needs of client is required, to justify the use of a frailty screening tool. (U) |  |  |
| The form screening should take and its message to older adults would be significant in terms of its utility in the prevention of frailty. (U) |  |  |
| Factors that should be included in screening (U)  1.a baseline for screening |  |  |
| Support for formal screening depends on its purpose and context (U) |  |  |
| Providers identified the importance of understanding the context in which frailty screening takes place. (U) |  |  |
| Given proper approaches to communicating, some regarded screening as potentially useful, particularly if it provided insight into what could happen in the future. (C) | 5.Constructing a trustful relationship |  |
| Providers also identified their role in providing information to older adults. Building a positive and trusting relationship between patient and provider is important for successful treatment and management. (U) |  |  |
| Frailty screening tool need to be presented in a sensitive manner(U) | 6.Conducting frailty screening by a sensitive approach |  |
| Frailty screening needs to be conducted sensitively screening pathway(U) |  |  |
| an integrated management approach of a MDT as integral to the management of frailty in the Primary care setting. (U) | 7. Involve the multidisciplinary team |  |
| older adults think multidisciplinary teams provide a good opportunity to screen for frailty based on their area of expertise. (U) |  |  |
| Lack of supporting evidence that proactive identification leads to improved patient care(U) | 8.Lack of support evidence of screening effectiveness | Synthesized findings 3: Motivation in the implementation of frailty screening  Healthcare providers’ positive attitude and the belief in the benefits of screening facilitate the implementation. Factors that hinder the implementation include the lack of supportive evidence of screening effectiveness, older adults’ fear of frailty, and doubt about community insufficient resources. |
| Overreach.  There were concerns highlighted by one GP that proactively identifying these patients was an overreach of the role of primary care (U) |  |  |
| Providers also describe how compliance in screening does not always mean patients are receiving the best care. Some providers felt that the time it took to assess a person frailty risk is time that could have been used more effectively helping patients. (U) |  |  |
| Acceptability of frailty screening(U) | 9.Positive attitude toward frailty screening among healthcare provider |  |
| Many GPs were positive about the idea of proactively identifying frailty. (U) |  |  |
| It is important to capture pre-frailty older adults to prevent them becoming frail. (U) |  |  |
| Providers described a desire to identify frailty and associated risks earlier, and treat concerns collaboratively with patients and other providers are appropriate. (U) |  |  |
| Subjective opinion versus objective score. (U) | 10.Healthcare providers perceived benefits of screening |  |
| frailty was considered subjectively at times, the introduction of a frailty screening tool formalized the assessment of frailty and made it a more prominent aspect of clinical practice among all older adults receiving physiotherapy. (U) |  |  |
| Some GPs acknowledged that recognizing frailty subjectively on the basis of visible physical indicators might result missed opportunities to address frailty earlier. (U) |  |  |
| Using the EFS as an objective screening tool of frailty enhanced their communication with the MDT. (U) |  |  |
| Frailty screening enhanced their communication with Family and Client. (U) |  |  |
| Frailty screening help client in a holistic Approach. (U) |  |  |
| Frailty screening increased their awareness of cognition. |  |  |
| Older adults regard frailty screening as something a person is unlikely to want to know the answer. (U) | 11. Older adults fear and escape frailty |  |
| Participants regarded frailty and frailty screening with fear and apprehension. And they may be not want to know whether or not they are frail. (U) |  |  |
| Providers discussed that patients often over-estimate their abilities. Older adults discussed this as hiding certain health related concerns from providers. Humour was often used to cover up concerns, providing a response to the question asked without providing a real answer. (U) |  |  |
| It would be expensive for health and social care systems to implement the kind of screening required, i.e. screening that is consultative, sensitive and which leads to personalized care. (U) | 12. Older adults question the community's insufficient resources |  |
| Older adults worrying the sufficient resources to provide services. (U) |  |  |
