# Supplementary material for: Perspectives of older adults, caregivers, healthcare providers on frailty screening in primary care: a systematic review and qualitative meta-synthesis
Source: BMC Geriatr. 2022 Jun 3;22:482. doi: 10.1186/s12877-022-03173-6 (PMC9166584; doi:10.1186/s12877-022-03173-6)
Supplement: Supplementary file 4 — Additional file 4. Recommendation for practice. [file 12877_2022_3173_MOESM4_ESM.docx]

**Additional file 4: Recommendation for practice**

| Recommendation | Joanna Briggs Institute Grade |
| --- | --- |
| We recommend provide frailty education and training for healthcare provider, older people and caregivers in primary care. | A |
| Healthcare providers should be encouraged to establish a trustful and positive relationship with older adults in primary care. | A |
| We recommend establish and integrate the multidisciplinary team to implement frailty screening in primary care. | A |
| We suggest that the government increase its investment for health resources in primary care. | B |
| We recommend healthcare providers conduct frailty screening by a sensitive approach. | A |
| We recommend healthcare providers notice the reaction of older adults to the negative term “frailty” and frailty screening. | A |
